# Custom Made Monoflange Acetabular Components for the Treatment of Paprosky Type III Defects

**DOI:** 10.3390/jpm11040283

**Published:** 2021-04-08

**Authors:** Sebastian Philipp von Hertzberg-Boelch, Mike Wagenbrenner, Jörg Arnholdt, Stephan Frenzel, Boris Michael Holzapfel, Maximilian Rudert

**Affiliations:** 1 Department of Orthopaedic Surgery, Orthopädische Klinik König-Ludwig-Haus, University of Wuerzburg, 97070 Wuerzburg, Germany; m-wagenbrenner.klh@uni-wuerzburg.de (M.W.); j-arnholdt.klh@uni-wuerzburg.de (J.A.); b-hozapfel.klh@uni-wuerzburg.de (B.M.H.); m-rudert.klh@uni-wuerzburg.de (M.R.); 2 Department of Orthopaedics and Trauma Surgery, Medical University of Vienna, Vienna General Hospital, 1090 Vienna, Austria; stephan.frenzel@meduniwien.ac.at

**Keywords:** patient specific implant, custom made implant, revision hip, Paprosky, pelvic discontinuity

## Abstract

Purpose: Patient-specific, flanged acetabular components are used for the treatment of Paprosky type III defects during revision total hip arthroplasty (THA). This monocentric retrospective cohort study analyzes the outcome of patients treated with custom made monoflanged acetabular components (CMACs) with intra- and extramedullary iliac fixation. Methods: 14 patients were included who underwent revision THA with CMACs for the treatment of Paprosky type III defects. Mechanism of THA failure was infection in 4 and aseptic loosening in 10 patients. Seven patients underwent no previous revision, the other seven patients underwent three or more previous revisions. Results: At a mean follow-up of 35.4 months (14–94), the revision rate of the implant was 28.3%. Additionally, one perioperative dislocation and one superficial wound infection occurred. At one year postoperatively, we found a significant improvement of the Western Ontario and McMaster Universities Arthritis Index (WOMAC) score (*p* = 0.015). Postoperative radiographic analysis revealed good hip joint reconstruction with a mean leg length discrepancy of 3 mm (−8–20), a mean lateralization of the horizontal hip center of rotation of 8 mm (−8–35), and a mean proximalization of the vertical hip center of rotation of 6 mm (13–26). Radiolucency lines were present in 30%. Conclusion: CMACs can be considered an option for the treatment of acetabular bone loss in revision THA. Iliac intra- and extramedullary fixation allows soft tissue-adjusted hip joint reconstruction and improves hip function. However, failure rates are high, with periprosthetic infection being the main threat to successful outcome.

## 1. Introduction

The revision burden after total hip arthroplasty (THA) will increase [1]. Acetabular bone loss is a major surgical challenge in revision THA (rTHA), particularly in re-revisions or after implant migration. Successful acetabular reconstruction with long-term component fixation requires sufficient primary stability for secondary osteointegration. A broad range of surgical strategies are available, of which the most popular are antiprotrusion cages [2], hemispherical or asymmetrical cups with intra- or extramedullary fixation [3,4,5] and modular, highly porous acetabular revision systems with and without metal wedges, buttress augments, and cage options [6,7]. However, it has not yet been defined which strategy should be considered as the benchmark [8].

Although custom made implants consume great organizational and financial resources, they are a further treatment option for large osseous defects that otherwise cannot be managed with standard implants. Based on computed tomography (CT), custom made acetabular components offer the surgeon the option to add metal sockets to the implant volume according to the defect of the hemipelvis, to adjust flanges for fixation devices to the remaining bone stock, and to plan the reconstruction of the hip center of rotation (COR) [9].

Most custom made acetabular components are designed as triflanges. These custom made triflange acetabular components (CTACs) are intended to “span the gap” by bridging the periacetabular defect and provide fixation options at the Os ilium, the Os pubis, and the Os ischium. However, these components were initially designed for the posterior approach, and the approach has to be relatively extensile to position all three flanges correctly. Consequently, results for these acetabular implants are highly variable [10].

At the study institution, high-grade acetabular bone defects are treated with different types of “off the shelf” acetabular reconstruction systems via the anterior but mainly the lateral or anterolateral approach. One of these systems relies on the combination of extra- and intramedullary iliac fixation using an iliac flange and an optional intramedullary press-fit stem and has proven good results in various studies [4,5]. However, there are defect situations in which “off the shelf implants” do not seem to be appropriate, for instance, in cases with significant loss of supportive bone from the anterior or posterior acetabular rim maybe with additional resorption of the dome. For these patients, a custom made monoflanged acetabular component (CMAC) seems warranted. The iliac flange is fixed to the gluteal surface of the ilium and can be positioned via the standard anterior or lateral approaches. The implant can be armed with an intramedullary press-fit stem for additional fixation.

In the following, we report on the patients who have been treated with this implant for reconstruction of the acetabulum after complicated rTHA.

## 2. Methods

### 2.1. Patient Selection

Approval for this retrospective study was given by the institution’s review board (Reference number 2016072801). We retrospectively identified 18 cases that underwent acetabular reconstruction with CMACs between January 2010 and December 2019 at our department. Three cases were excluded since the indication for CMAC was malignancy, and one patient died during CMAC implantation.

### 2.2. Implant

**Figure 1:**
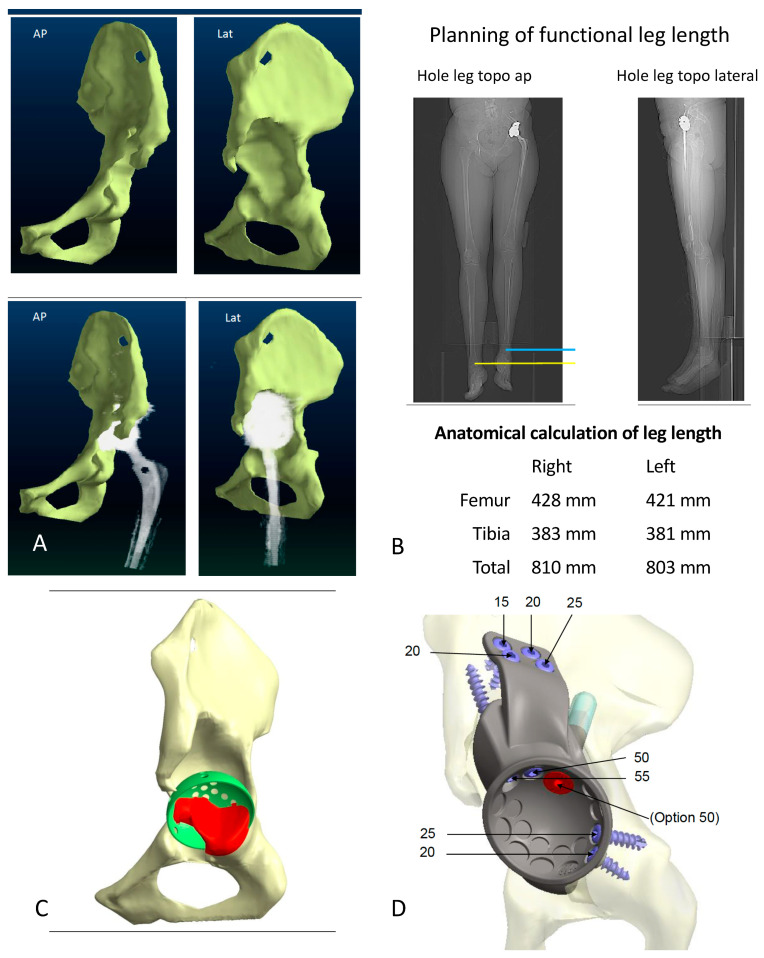
Selected templating steps for a custom made monoflanged acetabular component (CMAC) with optional stem for intra- and extramedullary iliac fixation for a Paprosky IIIA defect: (**A**) Assessment and 3D visualization of the defect situation with and without subtraction of the implant. (**B**) CT-based estimation of leg length discrepancy (LLD) respecting pelvic tilt and joint contractures. (**C**) Virtual reconstruction of the hip center of rotation (COR) by positioning a standard acetabular component of a specific size at the anatomical COR. Bone that has to be reamed to position the original implant is colored in red. (**D**) Design features of the CMAC: The large segmental iliac defect is filled by the implant’s metallic monoblock assembled socket. Screws are positioned in areas of the pelvis that are characterized by intact host bone with a recommendation for their length in millimeters. For further primary stability, the surgeon can implant an additional intramedullary press-fit stem (entrance point colored in red). Planning and defect classification have previously been described in detail by our group (11).

The CMAC is designed using data obtained via high-resolution CT imaging of the pelvis with an implant-specific algorithm (WinCad, Fa. AQ Solutions). Scans can be performed with or without a prosthesis or spacer in place. Figure 1 illustrates crucial templating steps that can be modified by the surgeon. After design approval by the surgeon, the implant is produced by laser melting of a titanium alloy (TiAI6V4) in a monoblock fashion with a 3D comb surface structure and with optional HA or CAP layering. The variability in form is reflected by Figures 1, 3 and 4. Manufacturing and delivering takes about 6 to 8 weeks.

### 2.3. Parameters Assessed

All presented data were extracted from the electronic patient charts. Preoperative acetabular defect situation was classified according to the modified Paprosky classification system based on preoperative radiographs and CT scans as described previously [11]. Postoperative radiographs were evaluated for reconstruction of the hip joint’s COR according to Rannawat [12]. Leg length discrepancy (LLD) was assessed by comparing the position of the trochanter minores to the connection line between Kohler’s teardrops.

Perioperative complications were defined as complication within 3 months after CMAC implantation and were tabulated as documented in the electronic patient chart. Implant revision after CMAC implantation was considered a failure. Failures were excluded from functional follow-up. Functional outcome was assessed using the Western Ontario and McMaster Universities Arthritis Index (WOMAC) Score that was recorded prospectively before and one year after CMAC implantation. Latest ap pelvic radiographs were examined for signs of implant loosening. Therefore, radiolucency lines thicker than 2 mm with sclerotic demarcation were considered significant [13]. Since the Charnley and DeLee zones are not applicable, 4 zones around the implant were defined: the cup, the metal socket, the iliac stem, and the iliac flange.

### 2.4. Patients

The cohort consisted of 14 patients, 5 men and 9 women. The operations were performed by hip and knee arthroplasty surgeons with additional specialization in revision cases. The operating surgeon indicated treatment with CMAC. Major decision criterion was bone loss at the ilium that did not enable adequate hip COR reconstruction with “off the shelf” cup and cage constructs or asymmetrical cups with intra- or extramedullary fixation. The mean age was 69.5 years (55–83), and the mean body mass index (BMI) was 28.0 kg/m^2^ (24.5–30.9), respectively. A total of 11 patients were classified as ASA III, and 3 patients as ASA II. Seven patients had no previous rTHA, the other 7 patients underwent 3 or more previous revisions. Indications for CMACs were spacer implantation after infection in 4 and aseptic loosening in 10 patients.

### 2.5. Statistics

Parameters are shown as mean and range. A Kaplan–Meier analysis for revision-free survival was performed. The Wilcoxon test was used to test paired samples for significance. A *p*-value <0.05 was assumed significant. Statistics were conducted with SPSS.

## 3. Results

Characterization of treated acetabular defects and treatment strategy is shown in Table 1. No additional osteosynthesis at the pelvis was performed during CMAC implantation.

### 3.1. Intraoperative Parameters

All patients were operated in supine position. A transgluteal Bauer approach was used in all but two patients, for which an anterolateral approach was more suitable. A semiconstrained liner was cemented into the acetabular construct in all but two patients who received a standard liner. Five patients underwent complete THA removal and spacer implantation before proceeding to CMAC implantation. The mean operation time for the seven patients with additional femoral stem exchange was 181 min (107–249). The mean operation time for the seven patients with only acetabular component exchange was 175 min (93–243). Postoperative weight bearing was restricted for 6 weeks in 11 patients and for 12 weeks in the remaining 3.

### 3.2. Perioperative Complications

One patient suffered from postoperative dislocation, which was managed with closed reduction. Another patient had a superficial wound infection that was managed with debridement. We did not observe perioperative fracture, nerve injury, or deep vein thrombosis.

### 3.3. Failures

**Figure 2:**
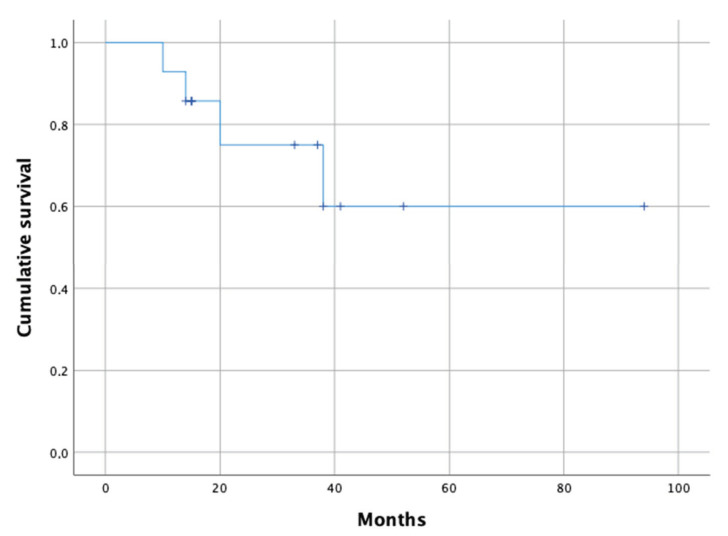
Kaplan–Meyer estimate of revision free-survival.

The mean follow-up was 35.4 months (14–94). We observed two acute septic failures (14.3%) at 10 and 35 months after CMAC implantation, which were treated with debridement, antibiotic therapy, irrigation, and implant retention. However, moving parts were exchanged in these two cases. Further, we observed two aseptic CMAC loosenings (14.3%). One patient was converted to a jumbo head after 14 months, and the other revised and the acetabular component replaced with a modular revision system 20 months after CMAC implantation. Figure 2 depicts the cumulative revision-free survival.

### 3.4. Function

Table 2 shows significant improvement of the WOMAC score and its subgroups pain and physical function in patients without failure one year after CMAC implantation. One patient did not complete the WOMAC questionnaires completely.

### 3.5. Radiographic Evaluation

**Figure 3:**
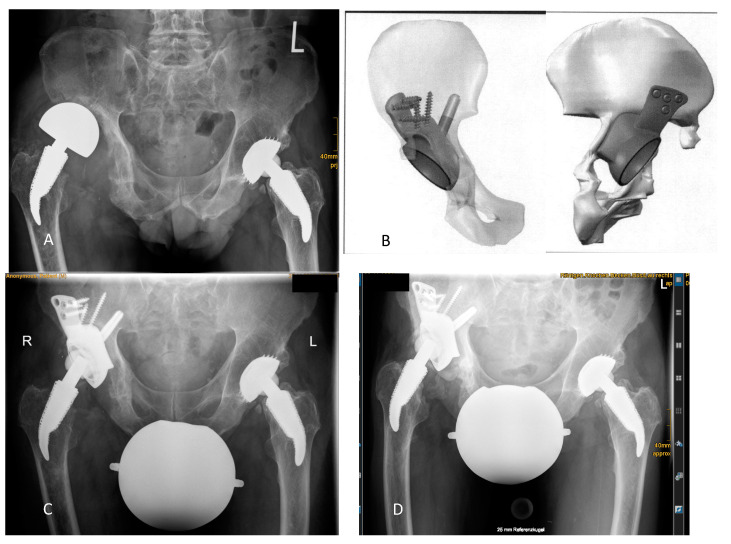
Radiolucency lines without need for revision: (**A**) Preoperative radiographic situation showed the acetabular “up-and-out” defect after implantation of a large head because of acetabular bone loss. (**B**) Anterior to posterior (left) and posterior to anterior (right) views show the intended proximalization of the COR in the virtual 3D reconstruction. The cup is not placed at the level of the Kohler’s tear drop. (**C**) Radiograph after revision and CMAC implantation showed restoration of leg length with a high COR. (**D**) Significant radiolucency lines developed around the whole implant at 3 years of follow-up. Although implant migration cannot be excluded, the patient was not revised because he reported daily walks of up to 10 km supported by a cane. Thus, this case was not considered a failure.

**Figure 4:**
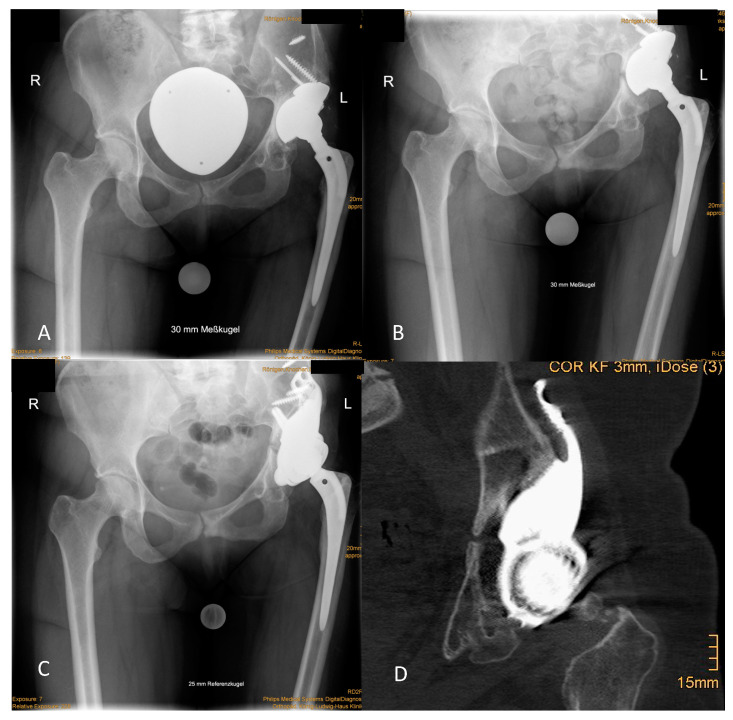
Osteointegration of the socket at follow-up. (**A**) Preoperative situation demonstrated an “up and out” defect that was filled by the loosened cup and augment construct. (**B**) The radiographic control after 2 years displayed PD with complete disruption of the ilio-ischial line and medial protrusion of the cup. (**C**) Radiographic situation 2 years after revision showed no sign of loosening. (**D**) In the CT, spot welds, as sign of osteointegration at the HA-coated socket, were seen.

Results of radiographic evaluation are shown in Table 3. Two patients were planned with intentional extra-anatomic reconstruction of COR as depicted in Figure 3 and Figure 4.

For the 10 patients without failure, significant radiolucency lines were found around the socket for one patient, around the acetabular construct for another patient, and around the whole CMAC for the last (Figure 3):

## 4. Discussion

Acetabular bone loss remains a major surgical challenge in complicated rTHA. With the presented CMAC we found acceptable results with significant improvement of function one year after implantation and an implant revision rate of 28.6% at a mean follow-up of 35.4 months.

The reported outcome is certainly influenced by patient-related presuppositions for acetabular reconstruction, which are anteceding or even subliminal infection and massive bone loss. In the current study, all patients had at least a Paprosky type III acetabular defect and 42.86% of patients even displayed PD. The optimal surgical strategy for those patients has not yet been defined. A stable pelvic ring is discussed as the “conditio sine qua non” for prevention of mechanical failure of acetabular constructs [7]. Antiprotrusion cages and CTACS as well as cup cage constructs aim to fulfill this strategy [14]. In contrast, the presented implant design abandons this strategy and relies on a combination of intra- and extramedullary iliac fixation for primary stability. However, positioning of the stem can be challenging. In two cases with a IIIa defect but with sufficient medial abutment by the remaining bone, the stems were dispensed. Implant loosening was not observed in these cases. However, whenever possible the iliac stem should by applied for optimal fixation. A rigid fixation of the CMAC to the Os ilium allows osteointegration as depicted in postoperative CT scans (Figure 4).

Irrespectively of the fixation strategy, component fixation seems to be rather successful while other complications are frequent. This statement is underlined by the literature and the data presented data here with high complication rates but acceptable acetabular component survival: In the review of CTACs by Chiarlone et al., the acetabular component survival rate ranged from 86.5% to 100%, but the reoperation rate was 24.5% [15]. In the review by De Martino et al., aseptic loosening of CTACs occurred in only 1.7%. However, the complication rate was 29% [10]. CTACs are designed to span the periacetabular gap by fixation to the iliac, the ischial, and the pubic bone. In contrast, Burastero et al. described a modular press-fit implant design with an antiprotrusion collar for patient-specific acetabular reconstruction and observed osteointegration of all implants at follow-up [8]. The acetabular component survival rate in the current study was 85.72%. However, overall complications occurred in 42.86%. This extremely high rate is comparable to the rate reported in the literature. De Martino et al. and Chiarlone et al. reported reoperation and complication rates ranging from 0 to 66.7% [10,15].

To the best of our knowledge, there is only one other study analyzing the outcome of CMACs. Walter et al. investigated and compared the outcomes of different designs of CTACs. With a mean follow-up of 79.8 months for the CTAC group and 43.0 months in the CMAG group, they found no significant difference regarding the implant survival rate, which was 28.6% and 21.6%, respectively [16].

In comparison to the three-point fixation for CTACs, the iliac fixation is advantageous because it requires less preparation at the ischium. Additionally, it can routinely be implanted in supine position, which facilitates leg length evaluation.

There are limitations to this study. Acetabular defects were assessed based on the preoperative templating CTs, instead of radiographs as initially described by Paprosky. This is warranted for the following reasons: First, radiographic evaluation is not feasible if the volume of the indwelling prosthesis covers bony landmarks. Second, PD does not always match the Paprosky classification [7] and finally, radiographic evaluation has demonstrated high inter- and intraobserver variability and tends to underestimate the acetabular defect situation [11,17]. However, it remains the most popular classification system for acetabular bone loss.

The mean follow-up reflects only the short-term outcome, and the number of patients is limited. Because this study focused on CMACs as revision implants, three patients were excluded due to malignancy. Although the revision burden is increasing, patients that do not meet the criteria for the treatment with an “off the shelf” acetabular revision system are still rare. This limitation is reflected by the overall small number of only 579 and 627 patients in the aforementioned reviews [10,15]. To the best of our knowledge, the current study reports the largest cohort study of patients treated for acetabular bone loss after rTHA failure with one special CMAC design.

Due to the retrospective design of this study, we cannot directly compare the results to those of CTACs. In our hands, the advantages of monoflange fixation are so convincing that we prefer it over the use of CTACs. However, we do observe a trend to highly porous cup-cage constructs with optional wedges and buttress augments. This is mainly based on the instant availability and intraoperative flexibility. However, the surgical strategy used and its success is still highly dependent on the surgeon’s skills and his/her experience with a particular implant. CMACs should be considered in cases with a high-grade acetabular defect situation, in which particularly cranial or caudal acetabular bone loss endangers successful reconstruction.

## 5. Conclusions

CMACs can be considered an option for the treatment of acetabular bone loss in rTHA. Preoperative CT-based 3D planning yields reproducible results for leg length and hip COR. The limited available data show that iliac intra- and extramedullary fixation allows soft tissue-adjusted hip joint reconstruction and improves hip function. However, failure rates are high with periprosthetic infection being the main threat to successful outcome.

## Figures and Tables

**Table 1:** Classification of acetabular defects with treatment strategy and failures (number = N; pelvic discontinuity = PD).

Paprosky Classification with and without PD	N	Iliac Stem N	Failure N (Mode)
IIIa	2	0	1 (infection)
IIIa with PD	5	5	1 (aseptic)
IIIB	5	5	0
IIIB with PD	2	2	2 (one each)
total	14	12	4

**Table 2:** Patient-reported function assessment with the Western Ontario and McMaster Universities Arthritis Index (WOMAC) score (number = N); * only complete pairs were included.

	Preoperative (N)	1 Year Postoperative (N)	*p* *
pain	51.00 (10–92) (10)	21.27 (10–70) (11)	0.038
stiffness	53.50 (10–100) (10)	28.18 (10–60) (11)	0.068
physical function	72.30 (30–94) (10)	32.36 (1–85) (10)	0.007
all	63.89 (27–82) (10)	29.45 (11–78) (11)	0.015

**Table 3:** Radiographic evaluation.

	Mean (Min to Max)
Leg Length Discrepancy in mm	+3 (−8 to 20)
Lateralization of COR in mm	+8 (−8 to 35)
Proximalization of COR in mm	+6 (−13 to 26)

## Data Availability

The datasets used and/or analyzed during the current study are available from the corresponding author on reasonable request.
